# Pituitary adenylyl cyclase activating polypeptide inhibits *gli1 *gene expression and proliferation in primary medulloblastoma derived tumorsphere cultures

**DOI:** 10.1186/1471-2407-10-676

**Published:** 2010-12-09

**Authors:** Joseph R Cohen, Daniel Z Resnick, Pawel Niewiadomski, Hongmei Dong, Linda M Liau, James A Waschek

**Affiliations:** 1 Semel Institute/Department of Psychiatry and Biobehavioral Sciences, David Geffen, School of Medicine, University of California at Los Angeles, Los Angeles, CA 90095 USA; 2 Graduate Neuroscience Interdepartmental Program, University of California at Los Angeles, Los Angeles, CA 90095 USA; 3 University of California at Berkeley, Berkeley, CA 94720 USA; 4 Division of Oncology, Stanford University School of Medicine, Palo Alto, CA 94305 USA; 5 Jonsson Comprehensive Cancer Center, David Geffen School of Medicine, University of California at Los Angeles, Los Angeles, CA 90095 USA; 6 Department of Neurosurgery, David Geffen School of Medicine, University of California at Los Angeles, Los Angeles, CA 90095 USA

## Abstract

**Background:**

Hedgehog (HH) signaling is critical for the expansion of granule neuron precursors (GNPs) within the external granular layer (EGL) during cerebellar development. Aberrant HH signaling within GNPs is thought to give rise to medulloblastoma (MB) - the most commonly-observed form of malignant pediatric brain tumor. Evidence in both invertebrates and vertebrates indicates that cyclic AMP-dependent protein kinase A (PKA) antagonizes HH signalling. Receptors specific for the neuropeptide pituitary adenylyl cyclase activating polypeptide (PACAP, gene name ADCYAP1) are expressed in GNPs. PACAP has been shown to protect GNPs from apoptosis *in vitro*, and to interact with HH signaling to regulate GNP proliferation. PACAP/*ptch1 *double mutant mice exhibit an increased incidence of MB compared to *ptch1 *mice, indicating that PACAP may regulate HH pathway-mediated MB pathogenesis.

**Methods:**

Primary MB tumorsphere cultures were prepared from thirteen *ptch1^+/-^/p53^+/- ^*double mutant mice and treated with the smoothened (SMO) agonist purmorphamine, the SMO antagonist SANT-1, the neuropeptide PACAP, the PKA activator forskolin, and the PKA inhibitor H89. Gene expression of *gli1 *and [^3^H]-thymidine incorporation were assessed to determine drug effects on HH pathway activity and proliferation, respectively. PKA activity was determined in cell extracts by Western blotting using a phospho-PKA substrate antibody.

**Results:**

Primary tumor cells cultured for 1-week under serum-free conditions grew as tumorspheres and were found to express PAC1 receptor transcripts. *Gli1 *gene expression was significantly reduced by SANT-1, PACAP and forskolin, but was unaffected by purmorphamine. The attenuation of *gli1 *gene expression by PACAP was reversed by the PKA inhibitor H89, which also blocked PKA activation. Treatment of tumorsphere cultures with PACAP, forskolin, and SANT-1 for 24 or 48 hours reduced proliferation.

**Conclusions:**

Primary tumorspheres derived from *ptch1^+/-^/p53^+/- ^*mice exhibit constitutive HH pathway activity. PACAP antagonizes HH signalling in these cells in a manner blocked by the PKA antagonist H89. PACAP and pharmacological activation of PKA also inhibited proliferation. Our data suggests that regulation of HH signaling by PACAP/PKA signaling may provide an alternative to SMO inhibition for the treatment of MB.

## Background

Medulloblastoma (MB) is a malignant pediatric brain tumor that is thought to arise in many cases from transformation of granule neuron precursors (GNPs) within the external granular layer (EGL) of the developing cerebellum reviewed in [1]. Medulloblastoma is classified into the following subtypes based on their phenotypic [2] and genetic characteristics [3]: classic, large cell, anaplastic, desmoplastic and MB with extensive nodularity. Analyses of human desmoplastic MB samples revealed altered activity of, and/or mutations in, molecules of the sonic hedgehog (HH) signaling pathway including *patched-1 *(*ptch1*) and *glioma-associated oncogene homolog 1 *(*gli-1*) [1,3-5]. HH signaling is pivotal in the regulation of CNS patterning and development [6-8] stem cell self-renewal [9] and MB pathogenesis [4,5,10], and has also been implicated in a variety of additional human cancers including lung, pancreatic, and prostate reviewed in [11]. Mutations in *ptch1 *(the gene that codes for the Patched-1 12 pass transmembrane protein, a negative regulator of the HH signaling cascade), are observed with Gorlin's (Basal Cell Nevus) Syndrome - an autosomal hereditary disease characterized by the development of MB, basal cell carcinomas, and rhabdomyosarcoma [12,13]. *Ptch1*^-/- ^mice die *in utero *while *ptch1^+/- ^*mice typically survive but develop cerebellar tumors closely resembling MB in about 15% of the cases [10].

Our present understanding of the HH signaling cascade suggests that binding of the hedgehog ligands to PTCH-1 de-represses smoothened (SMO), allowing for the cascade of events leading to the activation of cubitus interruptus (Ci) in flies and the Gli transcription factors in vertebrates reviewed in [14,17]. The Gli transcription factors bind to the promoters of several genes considered to be HH targets (including *gli1 *and *ptch1*) [16-19]. Presently, molecules that act directly on components of the canonical HH pathway, including several that target SMO, are in various stages of clinical trials [20]. On the other hand, the protein kinase A (PKA) signalling pathway is known to antagonize hedgehog (HH) signaling in both invertebrates [21] and vertebrates [22,23], suggesting an alternative approach to blocking overactive HH activity. Nonetheless, little is know about the significance of the PKA/HH interaction in the pathogenesis of MB and other tumors.

PACAP is a 38-amino-acid peptide originally identified on the basis of its ability to induce production of cAMP in pituitary cells [24]. PACAP regulates a variety of biological functions including neuroblast proliferation [25,26], and neuroblast survival [27]. In the developing brain PACAP binds to, and signals through, the G-protein coupled receptor PAC1, increasing cAMP production and PKA activity [28]. PAC1 is also coupled to other signaling cascades in some cells, including phospholipase C (PLC) [29], phosphatidylinositol 3-Kinase (PI3-K) [30], src [31], and the MAP kinase pathways [32-34].

Gene transcripts for the HH targets genes *ptch1 *and *gli1 *are expressed within the EGL in the developing rat and mouse cerebella [26,35], indicating that the HH pathway is active in these cells. PAC1 gene transcripts are also present in the EGL [26], suggesting that the PACAP and HH pathways might interact within the proliferating cells in this layer. Moreover, the robust proliferative action of SHH on cultured GNPs was completely blocked by either treatment with PACAP or pharmacological induction of cAMP/PKA [26]. The potential significance of a PACAP/HH interaction in MB was recently demonstrated. First, PAC1 gene transcripts were found to be abundantly expressed in MB preneoplastic lesions in *ptch1 *mutant mice [36]. Second, deletion of a single copy of PACAP in *ptch1^+/- ^*mutant mice was found to increase the incidence of MB approximately 2.5 fold [36]. Taken together, these data suggest that PACAP may regulate HH signaling in MB pathogenesis. In this work we investigate the interaction between HH and PACAP/PKA signaling in murine primary tumorspheres derived from MB tumors arising in *ptch1^+/-^*/*p53^+/- ^*double mutant mice. Our data suggests that regulation of HH signaling by PACAP/PKA may provide a novel alternative to SMO inhibition for the treatment of medulloblastoma (MB).

## Methods

### Animal Generation, Care, and Assessment of Medulloblastoma

These studies utilized mice with single copy mutations in both the *ptch1 *and the *p53 *genes. The creation of these mice has been reported previously [10,37]. Generation of *ptch1^+/-^/p53^+/- ^*mice were achieved by crossing *p53*^-/- ^and *ptch1*^+/- ^mice as previously described by Wetmore, Eberhart and Curran, 2001 [38]. Mice were housed according to UCLA Institutional Guidelines, including standard light-dark cycles with food and water ad libitum. All mice were monitored daily for evidence of tumor presence or illness.

### Drugs and Stock Concentrations

PACAP-38, SANT-1 (a HH pathway antagonist), purmorphamine (a HH pathway agonist), and H-89 (a PKA antagonist) were obtained from Calbiochem. DMSO (vehicle) and forskolin (a cAMP/PKA inducer) were obtained from Sigma. Treatment concentrations for the drugs, unless otherwise stated, were as follows: 10 nM PACAP, 5 μM forskolin, 3 μM purmorphamine, 1 μM SANT-1 and 30 μM H89. When DMSO was used as drug solvent its concentration was adjusted to 0.25% (v/v) for all samples, except for experiments where H89 was used, in which case the final DMSO concentration was adjusted to 0.55%. In all experiments using H89, cells were pre-treated with this agent 30 minutes prior to addition of other drugs.

### Isolation of Primary Tumorspheres, Maintenance, and Treatment

GNP-like tumor cells were isolated in a manner similar to that of Zhao, H et al., 2008 [39]. A total of thirteen individual tumor isolations and preparations were performed. Briefly, tumors were minced in 1 mL of trypsin/DNAse, placed into 1.5 mL eppendorf tubes and centrifuged at 750 × *g *for 5 min at 4°C. Pellets were resuspended in 800 μL of cold DNAse and immediately triturated with a 1000 μL filtered pipet, followed by trituration with a 200 μL pipet, and lastly with a fire polished Pasteur pipet. After another centrifugation as above, 500 μL of DNAse was added to the pellet with a trituration. This sample was resuspended in 4.5 ml of DMEM-F12 and applied to a 40 μm strainer (BD Falcon). The cell suspension was carefully layered on top of a 32%/60% percoll gradient and centrifuged at 2985 × *g *for 20 min at 4°C. Cells collected from the layer between the 32% and 60% percoll were washed once with cold DMEM-F12 by centrifuging at 2985 × *g *for 10 min. The supernatant was aspirated and the resulting pellet was resuspended in serum free medium comprising of DMEM/F12 medium (Invitrogen), Heparin [2 μg/mL](Sigma), Penicillin/Streptomycin/Glutamine [1%] (Invitrogen), bFGF [0.2 μg/mL] (Peprotech), EGF [0.02 μg/mL](Sigma), and B27 supplement [1:50] (Invitrogen). The cells were then counted and plated into 6-well plates containing 2 mL of medium such that the final cell concentrations were either 1 × 10^6 ^cells/mL or 0.5 × 10^6 ^cells/mL. Cells were then cultured for 6 or 7 days. During this period the cells were supplemented with additional bFGF (0.05 μg) and EGF (0.04 μg) on days 3 and 5. On day 6 or 7, serum-free media was added when necessary to equalize all volumes at 2 mL, and then drug treatments were added to the cells in the same wells as indicated in the figure legends.

### RNA Extraction and RT-PCR

Tumorspheres were pelleted in 1.5 mL eppendorf tube by microcentrifugation (750 × *g *for 5 minutes at 4°C). RNA was extracted from the samples using the TRIzol reagent (Invitrogen) according to manufacturer's instructions. cDNA was generated using the iScript cDNA synthesis kit (BioRad). Semi-quantitative RT-PCR for *pac1 *and SYBR-Green quantitative real-time RT-PCR for *gapdh *and *gli1 *were performed as described previously [26,36]. Individual sample differences were calculated based on the ratio of standard quantities of *gli1 *and *gapdh *transcripts determined by the standard curve method.

### Western Blotting

Whole-cell lysates, prepared in NP-40 lysis buffer along with 1 mM PMSF, 1 μg/ml each of aprotinin, leupeptin, and pepstatin, 1 mM Na_3_VO_4_, 1 mM NaF (Sigma), were analyzed by immunoblotting. Equal amounts of proteins from whole-cell extracts were resolved on sodium dodecyl sulfate-polyacrylamide gel electrophoresis (SDS-PAGE) and transferred to PVDF membranes. The membranes were subjected to Western blot analysis with rabbit anti-phospho-PKA substrate antibody (1:2000; Cell Signaling Technology) or mouse anti-β-actin antibody (1:5000; Cell Signaling Technology). The signals from the primary antibody were amplified by horseradish peroxidase-conjugated goat anti-rabbit or anti-mouse immunoglobulin G (Santa Cruz Biotechnology) and detected with enhanced chemiluminescence (GE Healthcare).

### Proliferation Studies

Thymidine incorporation in tumorsphere cultures was performed using the trichloroacetic acid (TCA) precipitation method. Dissociated cells isolated from tumors by percoll gradient as described above, were seeded in 12-well tissue culture plates at the initial density of 1×10^6 ^cells/well and cultured in serum free media as indicated above. After 24 hours, the tumorspheres were treated for either 24 or 48 hours with indicated drugs. [^3^H]-thymidine (NEN/PerkinElmer), 2 μCi/well, was added to the wells during the last 18 hours of treatment. Tumorspheres were harvested and centrifuged at 800 × g for 10 min at 4°C. After removal of the supernatant, the pellets were washed twice with 1 mL of PBS then lysed in 500 μl of NaOH (0.5 M) for 10 minutes at room temperature. Cell extracts were transferred into fresh tubes and DNA was precipitated with 500 μl of cold 20% TCA on ice for 20 min. The samples were centrifuged at 20,000 × g for 20 min at 4°C. Supernatants were aspirated and the DNA-containing pellets washed with 500 μl of 10% TCA. After a 10 min centrifugation, pellets were finally reconstituted in 500 μl of 0.1 M NaOH and transferred into scintillation vials. Incorporated radioactivity was counted on a scintillation counter (Beckman) using Aquasafe™ scintillation cocktail (Wallac).

### Statistics

Statistical analyses were performed using the GraphPad Prism 4.0 software. All *P *values were calculated using Student's t-test for comparison between two samples only, and ANOVA followed by Bonferroni's post hoc test for multiple comparisons. A value of *P *< 0.05 was considered significant. All data were normalized to vehicle controls.

## Results

### Primary Mouse Medulloblastoma Cells Grow as Tumorspheres and Exhibit Constitutive Hedgehog Pathway Activity

**Figure 1:**
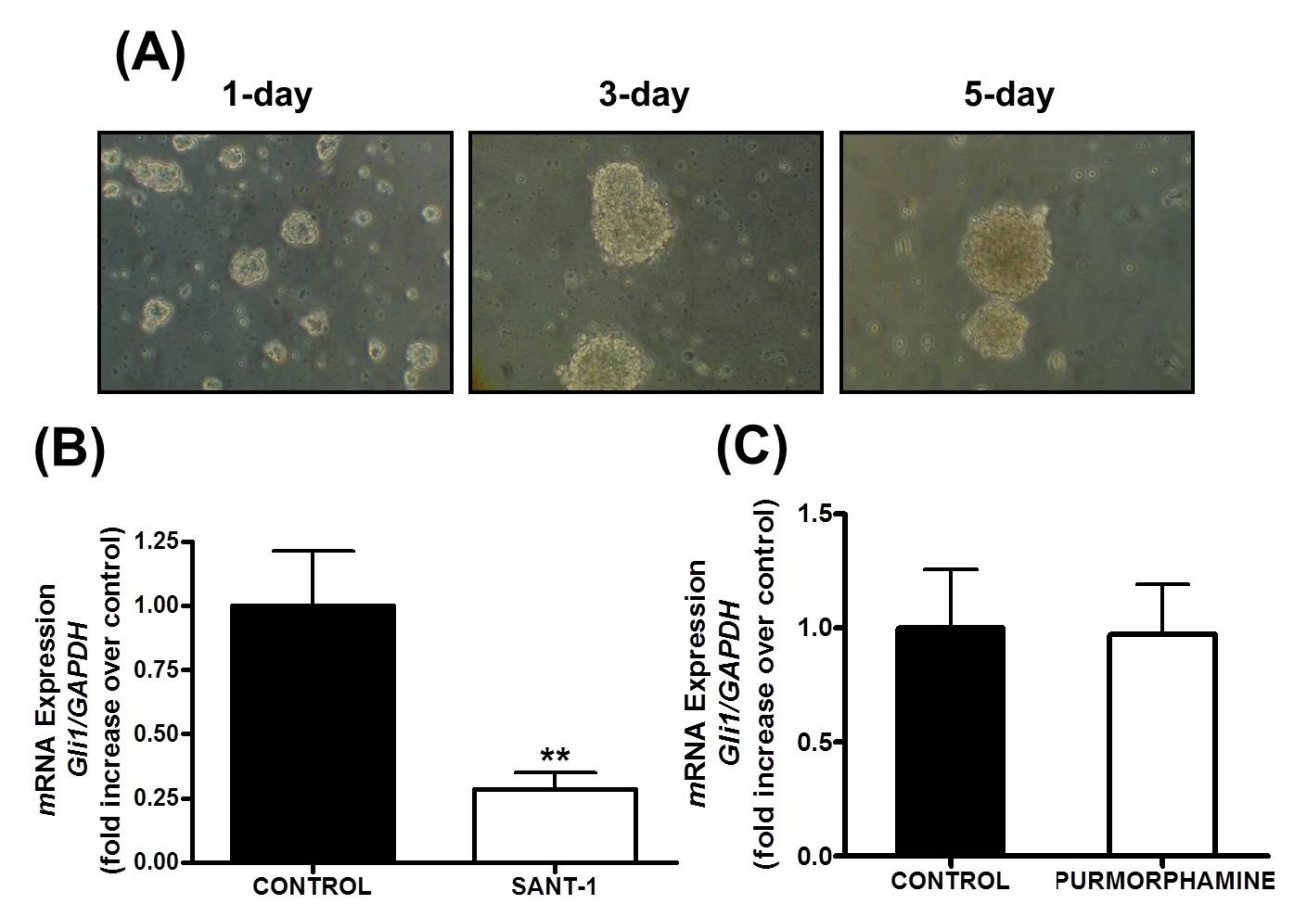
**Primary Medulloblastoma Cells Form Tumorspheres and Exhibit Constitutive Hedgehog Pathway Activity**. (**A**)Primary medulloblastoma cells from *ptch1^+/-^/p53^+/- ^*mice cultured in a serum-free environment formed tumorsphere-like aggregates within 24 hours, which became progressively larger at three and five days.(**B and C**) Quantitative real time RT-PCR analysis of *gli1 *gene expression in 6-7 day old tumorsphere cultures treated for 6 h with either the smoothened antagonist SANT-1 (1 μM) **(B) **or the smoothened agonist purmorphamine (3 μM) **(C) **Gene expression experiments were performed at least three times and with at least n = 3 samples for each treatment. (**P < 0.01).

Cell lines derived from human or murine MB tumors have been used to understand the biology of MB, but recent evidence indicate that propagated MB cell lines lose much or all of their responsiveness to HH pathway antagonism [40]. We therefore harvested fresh primary MB tumor cells for each experiment. We utilized *ptch1^+/-^/p53^+/- ^*double mutant mice for our studies because mutations in the *p53 *tumor suppressor gene in the context of *ptch1 *haploinsufficiency results in a higher incidence of MB than that in mice with the *ptch1 *mutation alone [38]. Tumor cells were harvested and purified by percoll gradient, and single cell suspensions were plated in serum-free media for 1 week using a modification of methods reported by others to preserve HH pathway-dependent proliferation [39]. After several days of culture the cells formed aggregates of increasing size, referred to as "tumorspheres" (Figure 1A). To determine if the HH pathway was active in these cells, we treated these cultures at the end of the 1-week period with the HH pathway antagonist SANT-1 (a known inhibitor of smoothened (SMO), 1 μM [41]) and measured the expression of the HH target gene *gli1 *by real time RT-PCR. SANT-1 significantly decreased *gli1 *expression (Figure 1B) relative to vehicle. These data implied that the HH pathway is constitutively active in tumorsphere cultures generated using our experimental paradigm.

To determine if the HH pathway could be activated beyond basal levels in these tumorsphere cultures, we treated them with the SMO agonist purmorphamine at a concentration (3 μM) shown to induce maximum HH signalling without cellular toxicity [42-44]. Purmorphamine failed to induce *gli1 *expression above control levels, suggesting that the HH pathway was already maximally activated in the tumorspheres under these experimental conditions (Figure 1C).

### Pituitary adenylyl cyclase activating polypeptide (PACAP) and Forskolin Inhibit Hedgehog Pathway Activity in Primary Tumorspheres

**Figure 2:**
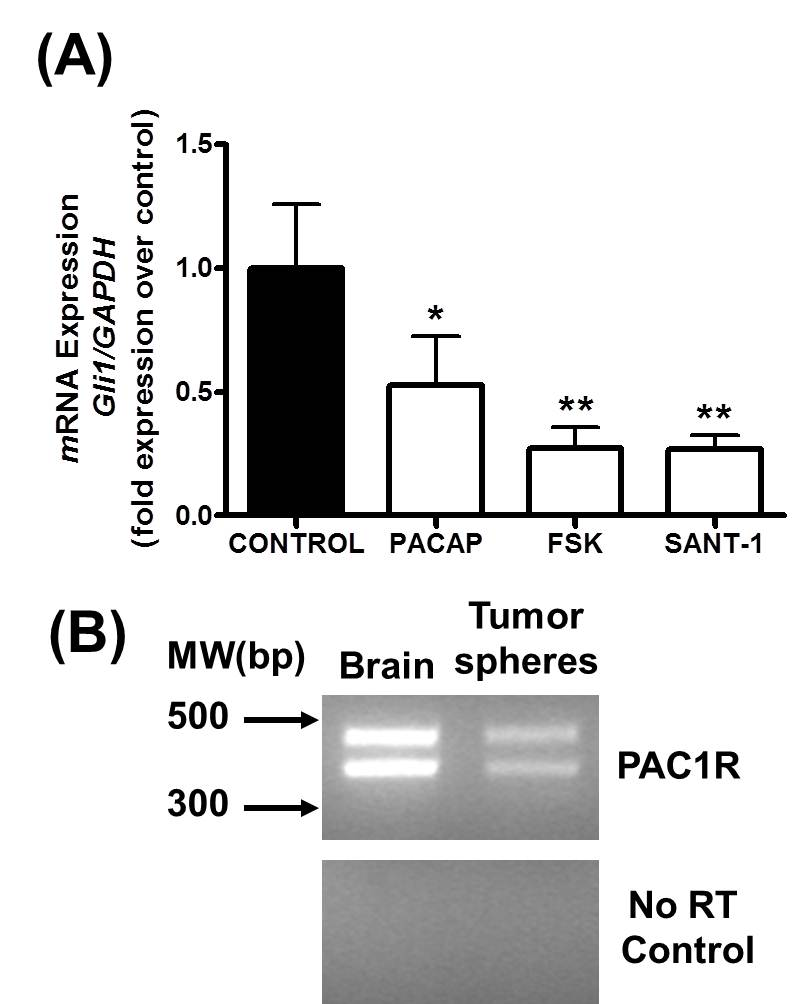
**PACAP and Forskolin Inhibit HH pathway activity in Primary Tumorspheres**. (**A**) Expression of the HH target gene *gli1 *in 6-7 day old primary tumorspheres treated for 6 hours with either vehicle, 10 nM PACAP, 5 μM forskolin, or 1 μM SANT-1, determined by real time RT-PCR. Experiments were performed three times with at least n = 3 for each treatment (*P < 0.05, **P < 0.01). (**B**) RT-PCR analysis demonstrating gene expression of the PAC1 PACAP receptor in primary tumorspheres. Total brain (left lane) was used as a positive control. Negative controls (lower panel) utilized the same samples, but were not subjected to reverse transcriptase (RT).

Because the HH pathway exhibited a significant level of constitutive activity in primary tumorsphere cultures, we investigated if this activity could be inhibited by either PACAP (10 nM) or forskolin (5 μM), an activator of adenylyl cyclase. We first performed RT-PCR analysis for the PACAP receptor PAC1 and confirmed that its gene expression is preserved in tumorspheres cultures (Figure 2B). Treatment with 10 nM PACAP and 5 μM forskolin resulted in significant down-regulation of *gli1 *in these cultures compared to vehicle treated controls (Figure 2A).

### Attenuation of Gli1 Expression in Primary Tumorspheres by PACAP was Associated with PKA Activation and was Reversed by the PKA Inhibitor H89

**Figure 3:**
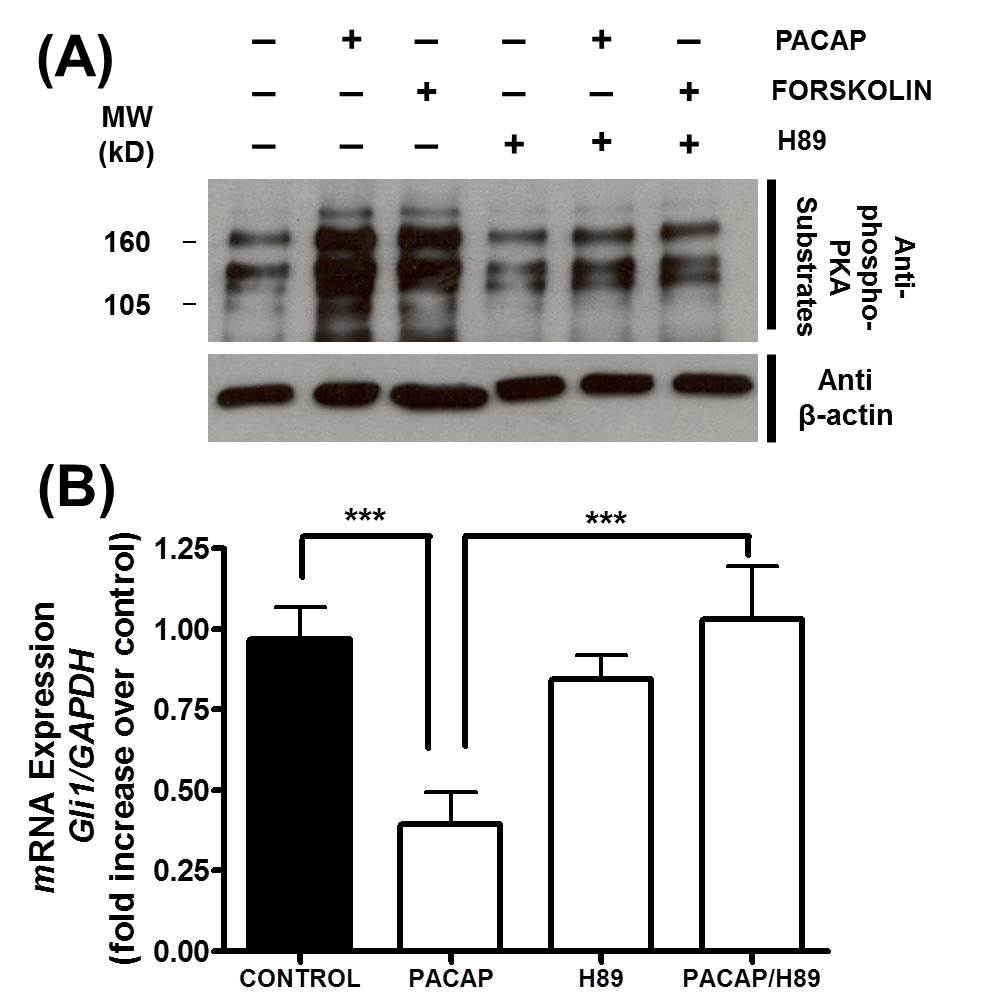
**Attenuation of Gli1 Expression in Primary Tumorspheres by PACAP was Associated with PKA Activation and was Reversed by the PKA Inhibitor H89**. (**A**) Western blot analysis of PKA activity in tumorsphere cultures treated with vehicle, 10 nM PACAP, 5 μM forskolin, 30 μM H89, 10 nM PACAP plus 30 μM H89 or 5 μM forskolin plus 30 μM H89. Blot was incubated with a rabbit phospho-PKA-substrate antibody (top) or a mouse β-actin antibody (bottom). The blot shown is representative of 3 experiments. (**B**) *Gli1 *gene expression in primary tumorspheres treated with either vehicle, 10 nM PACAP, 30 μM H89, or the combination of PACAP and H89. Experiments were performed at least 3 times and with at least n = 3 for each treatment (***P < 0.001).

As both PACAP and forskolin typically exert their effects in a PKA dependent manner, we investigated if their effects could be reversed by the PKA inhibitor H89. To confirm their effects on PKA activity, we performed Western blot analyses using an antibody that detects proteins containing a phospho-serine/threonine residue with arginine at the -3 and -2 positions, which are typically found on proteins phosphorylated by PKA. We observed that treatment of primary tumorspheres with either 10 nM PACAP or 5 μM forskolin induced similar increases in PKA activity (Figure 3A) compared to vehicle treated controls. Treatment with H89 (30 μM) reversed the inductions of PKA activity by these treatments (Figure 3A). Moreover, treatment with H89 reversed the reduction in *gli1 *expression by 10 nM PACAP (Figure 3B). These data indicate that PACAP attenuates HH signaling in primary tumorspheres, most likely via a PKA dependent mechanism.

### PACAP and Forskolin Inhibit Cellular Proliferation in Primary Tumorspheres

**Figure 4:**
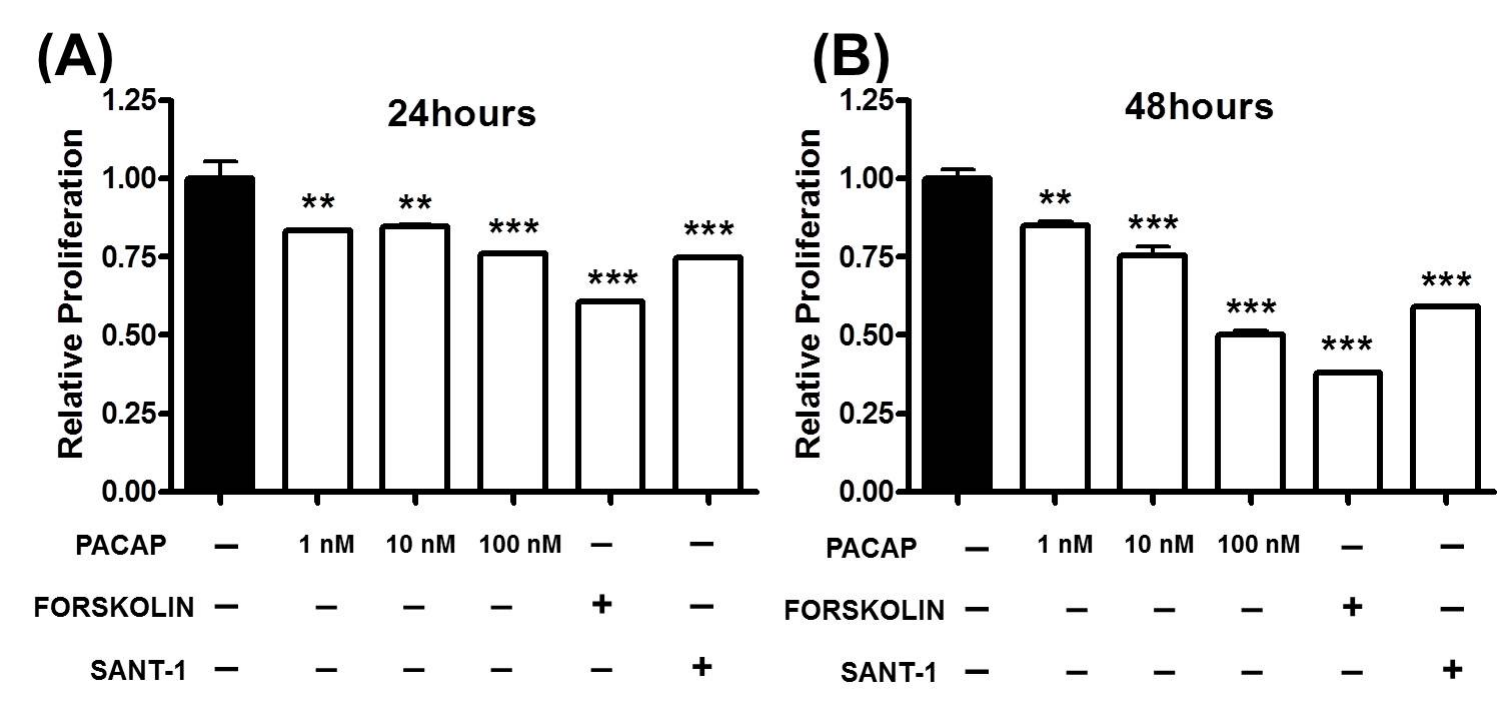
**PACAP and Forskolin Inhibit Proliferation in Primary Tumorsphere Cultures**. [^3^H]-thymidine incorporation assays were performed in cultures treated with vehicle, increasing concentrations of PACAP, 5 μM forskolin, or 1 μM SANT-1 for 24 hours (**A**) or 48 hours (**B**). In each case, [^3^H]-thymidine was added for the last 18 hr of the experiment. Experiment was performed three times, with 3-4 samples for each treatment (** P < 0.01, *** P < 0.001).

The above studies suggest that PACAP treatment or stimulation of PKA activity by other methods might provide strategies to inhibit the growth of MB tumors. We thus investigated the effects of PACAP (10 nM) and forskolin (5 μM) on cellular proliferation using [^3^H]-thymidine to measure DNA synthesis. As predicted, proliferation was reduced by 24 or 48 hour treatment with PACAP in a dose dependent manner. Forskolin (5 μM) and SANT-1 (1 μM), also decreased proliferation at these time periods (Figures. 4A and 4B). These data suggest that PACAP and PKA activation might provide therapeutic alternatives to SMO antagonism in the treatment of MB.

## Discussion

A large and diverse array of cancer types show evidence of overactive HH signalling [11]. This has triggered an intensive search for agents that selectively inhibit this pathway [20]. As a result, several molecules have been discovered or designed that directly interact and inhibit the activity of SMO, some of which are in clinical trials [20]. Despite the promise of this approach, two potential problems are that 1) some tumors acquire mutations in SMO that render them relatively resistant to SMO antagonists [45], and 2) SMO is critically involved in the growth of bones and other tissues [11,46], and appears to regulate stem cell biology [9] and [47]. Thus, there is a need for molecules that act downstream of SMO in the HH signalling pathway and that inhibit HH signalling specifically in tumor cells. PKA is thought to act in the HH pathway downstream of SMO, phosphorylating and triggering proteosome-mediated degradation of Gli2 and Gli3 [48,50]. Thus PKA activators might be expected to block HH signalling even in the context of drug-resistant SMO mutations. However, given that PKA is used in so many biological processes, it is unlikely that general activation of PKA will be useful as a treatment strategy. An alternative strategy would be to activate PKA specifically in tumor cells. This could potentially be achieved by targeting appropriate heterotrimeric G protein-coupled receptors expressed on the cells. The data reported here suggest that a potentially-effective strategy to treat MB tumors exhibiting overactive HH signaling would be to activate PACAP receptors. We investigated this possibility by using genetically engineered mice that spontaneously develop MB, and a tissue culture system that preserves a significant level of constitutive HH activity in tumor cells. We found that PACAP inhibited proliferation and the expression of the HH target gene *gli1 *in these cells. Moreover, the reduction of *gli1 *gene expression by PACAP was reversed by PKA inhibition, and was mimicked by stimulation of the cAMP/PKA pathway by forskolin. Overall, the data suggest that PACAP, via activation of PKA, inhibits HH signalling and HH-driven proliferation in MB cells. However, our findings do not rule out alternative mechanisms of PACAP/PKA action in these assays. For example, despite its action on *gli1 *in MB tumorspheres, the ability of PACAP to inhibit proliferation might be unrelated to its effects in HH pathway activity. In this respect, PACAP (and PKA activation in general) is known on inhibit the proliferation of many types of cells [51,53], some of which might not exhibit HH pathway activity. In any case, the findings reported here suggest that PACAP or PAC1 agonists could be useful as alternatives to, or in conjunction with, SMO antagonists, for the treatment of MB and other HH dependent tumors.

## Conclusions

Primary murine medulloblastoma derived tumorspheres from *ptch1^+/-^/p53^+/- ^*mice, cultured in a serum-free environment, express the PACAP receptor PAC1, and exhibit constitutive HH pathway activity as evidenced by sensitivity to HH pathway antagonist SANT-1. Treatment of tumorspheres with PACAP or the cAMP/PKA activator forskolin significantly reduces *gli1 *expression in a manner that is reversed by the PKA inhibitor H89. PACAP and forskolin also block tumorsphere proliferation. These finding suggest that PACAP or PKA activation may be useful for therapeutic intervention in the treatment of MB.

## Competing interests

The authors declare that they have no competing interests.

## Authors' contributions

JRC: designed and performed the experiments, analyzed and interpreted the data, and wrote the manuscript; DZR: helped in performing experiments; PN: helped with experimental design, technical expertise and data interpretation; HD: performed all animal care, husbandry, and genotyping; LML: helped with experimental design and interpretation of the data; JAW: coordinated the design and execution of experiments, supervised and participated in data analysis, and interpretation, and editing of the manuscript. All authors have read and approved the final manuscript.

## Pre-publication history

The pre-publication history for this paper can be accessed here:

http://www.biomedcentral.com/1471-2407/10/676/prepub
